# Commentary: Patient Preferences and Satisfaction of Nipple Areola Reconstruction with Three-Dimensional Tattoo in the Setting of Bilateral Implant-Based Breast Reconstruction

**DOI:** 10.1177/22925503251336144

**Published:** 2025-05-05

**Authors:** Karanvir S. Raman, Tyler Safran, Joshua Vorstenbosch

**Affiliations:** 1 Division of Plastic & Reconstructive Surgery, 5620 McGill University, Montreal, QC, Canada

The creation of the new nipple-areolar complex (NAC) is one of the final steps in breast reconstruction. Although we study breast reconstruction heavily, patient perspectives of the reconstructed NAC are often overlooked despite the significant value patients assign to NAC reconstruction yielding a major knowledge gap.^1,2^ In this study by Jones et al, the authors explore patient preferences related to size and position of NAC tattoos, variables contributing to their preferences, and BREAST-Q scores to quantify patient-reported outcomes.^3^

Traditionally, NAC placement in reconstruction has followed established metrics, including an areola diameter of 38 to 45 mm and a sternal notch-to-nipple distance of 21 to 22 cm.^4,5^ These standards, however, stem from analyses of select ideal native breast anatomy and aesthetic ideals in predominantly nononcologic populations.^6,7^ As such, there is growing recognition that patient-specific factors, such as body habitus and ethnicity, should play a greater role in determining NAC size and position.^7^ Additionally, the variability of the mastectomy scar, with transverse incisions generally allowing more adjustments than wise patterns, must be considered.^8,9^

Here, Jones et al provided valuable insights into patient preferences for three-dimensional (3D) NAC tattooing in bilateral implant-based reconstruction with transverse mastectomy scars. The study assessed 104 patients, allowing them to choose both the NAC tattoo size and location. Key findings include a preference for smaller areola diameters (average 3.62 ± 0.45 cm) and higher NAC positions compared to traditional standards, with a sternal notch-to-nipple distance of 19.53 ± 2.66 cm. This was also found to be the case in patients where 3D imaging was available for preoperative comparison of parameters (*P* < .001). Notably, larger implant size and Asian ethnicity were associated with larger areola diameters, while larger BMI, when adjusted for nipple-to-inframammary fold distance, was not associated with lower NAC placement. Importantly, they demonstrate a significant improvement in sexual well-being posttattoo, as measured by the BREAST-Q scores.^10^ These findings align with prior studies showing that tattoo-based NAC reconstruction enhances body image, particularly in intimate settings.^11,12^

The study's strengths include its largely homogenous cohort of alloplastic oncologic breast reconstructions and the use of 3D imaging for precise measurements adding important data to our understanding of patient preferences for NAC reconstruction. However, some factors warrant consideration. The study included only 2 Asian patients, limiting the generalizability of its findings despite reaching statistical significance. Additionally, while focused on 3D tattooing, it would be interesting to see how the data may change in nipple reconstruction with local flaps, where nipple projection may influence perceived NAC diameter.^7^ Other factors, such as mastectomy incision type (eg, transverse), its location relative to the desired NAC, and the impact of preexisting ptosis with age, would also be interesting to explore and see how these variables may affect NAC placement and patient satisfaction.^7^ Future studies investigating these points as well as exploring NAC placement patient preferences in autologous breast reconstruction may help to even better understand NAC reconstruction in breast cancer patients.

We commend the authors for their thorough and excellent study that adds to our understanding of patient preferences in NAC reconstruction, a very under investigated aspect of breast cancer reconstruction.
